# Evaporation Kinetics Engineering Suppressed Bimolecular Recombination in All‐Polymer Solar Cells

**DOI:** 10.1002/advs.77114

**Published:** 2026-08-17

**Authors:** Dinglong Feng, Le Mei, Chao Li, Yuang Fu, Heng Liu, Luhang Xu, Pok Fung Chan, Zhixiang Yang, Yiming Wang, Chun‐Jen Su, U‐Ser Jeng, Patrick Wai‐Keung Fong, Lijian Zuo, Gang Li, He Yan, Jiaying Wu, Xian‐Kai Chen, Xinhui Lu

**Affiliations:** ^1^ Department of Physics The Chinese University of Hong Kong Hong Kong People's Republic of China; ^2^ Institute of Functional Nano & Soft Materials (FUNSOM) Joint International Research Laboratory of Carbon‐Based Functional Materials and Devices State Key Laboratory of Bioinspired Interfacial Materials Science Soochow University Suzhou People's Republic of China; ^3^ Department of Chemistry and Hong Kong Branch of Chinese National Engineering Research Center for Tissue Restoration and Reconstruction Hong Kong University of Science and Technology Hong Kong People's Republic of China; ^4^ Hangzhou International Innovation Institute Beihang University Hangzhou People's Republic of China; ^5^ Advanced Materials Thrust, Function Hub The Hong Kong University of Science and Technology (Guangzhou) Guangzhou People's Republic of China; ^6^ State Key Laboratory of Silicon Materials MOE Key Laboratory of Macromolecular Synthesis and Functionalization Department of Polymer Science and Engineering Zhejiang University Hangzhou People's Republic of China; ^7^ National Synchrotron Radiation Research Centre Hsinchu Science Park Hsinchu Taiwan; ^8^ Department of Chemical Engineering & College of Semiconductor Research National Tsing Hua University Hsinchu Taiwan; ^9^ Department of Electrical and Electronic Engineering The Hong Kong Polytechnic University Hong Kong People's Republic of China; ^10^ Department of Electrical and Electronic Engineering Research Institute of Smart Energy (RISE) Photonic Research Institute (PRI) The Hong Kong Polytechnic University Hong Kong People's Republic of China

**Keywords:** acceptor, amorphous solid, chemical engineering, crystallinity, crystallization, materials science, polymer, spectroscopy, ternary operation

## Abstract

All‐polymer solar cells (all‐PSCs) combine the synthetic tunability of non‐fullerene acceptor systems with intrinsic mechanical and thermal robustness of polymer active layers, yet their performance remains limited by pronounced bimolecular recombination, leading to insufficient fill factors (FFs) compared to polymer:small‐molecule counterparts. Here, we introduce Phenanthrene (Ph) as a donor‐selective morphology regulator in the benchmark all‐PSC system. Capacitance spectroscopy reveals that bimolecular recombination dominates the recombination landscape and that Ph nearly halves the Langevin reduction factor, resulting in substantial improvement in FFs. Multiscale characterization, together with in situ optical spectroscopy and all‐atom molecular‐dynamics simulations, collectively show that, in contrast to 1‐chloronaphthalene (1‐CN, which remains in the film) and 2‐methylnaphthalene (2‐MN, which evaporates rapidly), Ph exhibits moderate evaporation kinetics during annealing. This enables enhanced donor crystallinity together with sufficient time and space for acceptor crystallization into fractal networks with increased fractal dimension, thereby reducing donor‐acceptor interfacial area. As a result, Ph‐assisted binary and ternary all‐PSCs achieve power conversion efficiencies of 18.74% and 19.35%, respectively. By explicitly correlating additive molecular structure and evaporation kinetics with multiscale morphology and recombination pathways, this work establishes rational selection rules for volatile solid additives and provides a general strategy to suppress bimolecular recombination in high‐performance all‑PSCs.

## Graphical abstract

In this study, phenanthrene (Ph), a volatile solid additive with a weak tendency toward self‐aggregation, is introduced as a donor‐selective morphology regulator in benchmark all‐PSC systems. The moderate evaporation kinetics of Ph enable cooperative crystallization of donors and acceptors, leading to an increased fractal dimension with reduced donor‐acceptor interfacial area, and thereby suppressed bimolecular recombination.

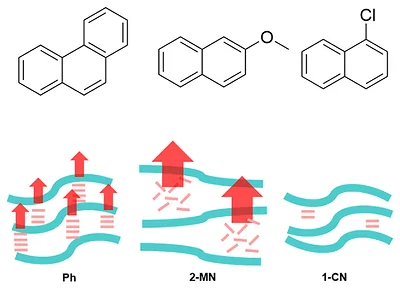

## 1 Introduction

Organic solar cells (OSCs) have witnessed remarkable advancements over the past decade, with certified power conversion efficiencies (PCEs) now exceeding 20%, driven largely by the development of small‐molecule non‐fullerene acceptors [1, 2], optimized charge transport layers [3, 4], and morphology control strategies [5, 6]. On the other hand, all‐polymer solar cells (all‐PSCs), which employ conjugated polymers as both donor and acceptor, stand out by combining the strong absorption and tunable bandgap of non‐fullerene acceptors (NFAs) [7, 8] with the inherent thermal tolerance [9] and mechanical robustness [10] of polymeric active layers, making them particularly attractive for large‐area manufacturing and flexible applications.

Despite these advantages, state‐of‐the‐art all‐PSCs still lag behind polymer:small‐molecule systems in fill factors (FFs) and overall PCEs. A major challenge in fabricating high‐performance all‐PSCs is controlling bulk‐heterojunction morphology across multiple length scales [7, 11, 12]. Relative to small molecules, the large molecular weight and chain entanglement of conjugated polymers lead to slow diffusion [13], while broad molecular‐weight distributions frustrate self‐assembly within domains [14, 15], and fast drying of low‐boiling‐point solvents (e.g., chloroform) can freeze non‐optimal phase separation [16]. These kinetic constraints often result in either overly mixed donor‐acceptor (D:A) morphologies, which enhance carrier encounter probability and promote bimolecular recombination, or overly coarse phase separation, which limits exciton dissociation and charge collection.

Additive engineering has emerged as one of the most effective strategies to tune phase separation, crystallinity, and interfacial area in all‐PSCs [17, 18, 19, 20, 21, 22, 23, 24]. In particular, additives with fused aromatic cores [22, 23, 24] have attracted extensive attention, leveraging their planar aromatic backbones to direct polymer packing through strong *π–π* interactions. For example, the liquid additive 1‐chloronaphthalene (1‐CN) is often employed to enhance domain purity and carrier mobility [25, 26], but its high boiling point and low vapor pressure tend to leave residual additive in the active layer, compromising long‐term stability and batch‐to‐batch reproducibility [27]. In contrast, solid naphthalene‐based additives (e.g., 2‐methylnaphthalene (2‐MN), 2‐ethylnaphthalene (2‐EN), 2‐phenylnaphthalene (2‐PN)) can be cleanly removed during thermal annealing, and have recently pushed all‐PSC PCEs to 19.5% [13, 19]. The target solid additive, phenanthrene (Ph), which we employed in this work, comprises three fused benzene rings in a bent “V” configuration [28], with strong sublimating property, which provides stronger morphology‐tuning capability than naphthalene cores and allows volatilization within typical annealing windows. Ph has been reported to boost the PCEs of polymer:NFA system PM6:Y6 devices above 19% [29, 30], but has not previously been employed in all‐PSCs. Nevertheless, additive selection has largely remained empirical, and the relationships among additive molecular structure, evaporation kinetics, morphology evolution, and specific recombination pathways, especially bimolecular recombination in all‑PSCs, are not yet systematically understood.

In this study, we demonstrate that evaporation kinetics represent a critical, yet underappreciated control parameter for suppressing bimolecular recombination in all‐PSCs. Using the benchmark PBQx‐TCl:PY‐IT all‐PSC system [31, 32, 33], we first reveal that bimolecular recombination dominates device losses, as evidenced by capacitance spectroscopy and the extracted Langevin reduction factor (*γ*). Multiscale structural characterization combined with in situ optical spectroscopy and all‐atom molecular‐dynamics simulations shows that Ph, with moderate volatility and weak self‐aggregation, selectively enhances donor crystallinity, increases nanophase domain size, and raises the fractal dimensionality of crystalline acceptor networks, thereby reducing the effective donor‐acceptor interfacial area. This structural evolution directly leads to a substantial reduction of *γ*—nearly by half compared to control additives—while maintaining low trap densities. Consequently, Ph‐assisted devices achieve FFs up to 75.9% and 76.7%, with PCEs up to 18.74% and 19.35% for binary and ternary devices, respectively. By explicitly correlating additive molecular structure with multiscale morphology and recombination mechanisms, this work establishes general selection rules for volatile solid additives and provides a promising pathway toward closing the performance gap between all‐polymer systems and polymer:NFA systems.

## 2 Result and Discussion

### 2.1 Device Performance and Recombination Mechanism

The chemical structures of the polymer donor PBQx‐TCl, polymer acceptor PY‐IT, and the three additives (Ph, 2‐MN, 1‐CN) are shown in Figure 1a. Solar cells were fabricated with a conventional device structure of ITO/2PACz/active layer/PNDIT‐F3N/Ag. Solid additives (Ph or 2‐MN) were dissolved in chloroform at 7 mg mL^−1^, while the liquid additive 1‐CN was used at 0.2 vol%; detailed fabrication procedures are provided in the **Experiment Methods** section. Representative *J‐V* curves and device statistics are shown in Figure 1b,c, and Figure S1, and the corresponding photovoltaic parameters are summarized in Table 1. The additive‐free PBQx‐TCl:PY‐IT device (BHJ‐no) delivers a PCE of 17.13% with *J*_SC_ of 25.43 mA cm^−2^, *V*_OC_ of 0.963 V, and FF of 69.4%. Upon incorporation of Ph, the optimized device (BHJ‐Ph) achieves a PCE of 18.74% with *J*_SC_ of 26.01 mA cm^−2^ (EQE in Figure S2), *V*_OC_ of 0.953 V, and a markedly enhanced FF of 75.9%. In contrast, devices processed with 2‐MN or 1‐CN exhibit more modest FFs of 70.7% and 71.5%, respectively. Moreover, BHJ‐Ph exhibits the most advanced thermal stability with *T*_80_ (aging time where PCE drops to 80% of the initial value) over 300 h and retained 95.5% initial PCE in the light‐soaking aging [34], together with improved ambient storage stability (Figure S3). The Ph‐assisted strategy was further extended to the ternary PBQx‐TCl:PY‐IT:PY‐DT‐X [19] system, yielding a champion PCE of 19.35%, with *J*_SC_ of 26.79 mA cm^−2^ (Table S1, EQE in Figure S4), *V*_OC_ of 0.945 V, and FF of 76.7%.

**FIGURE 1:**
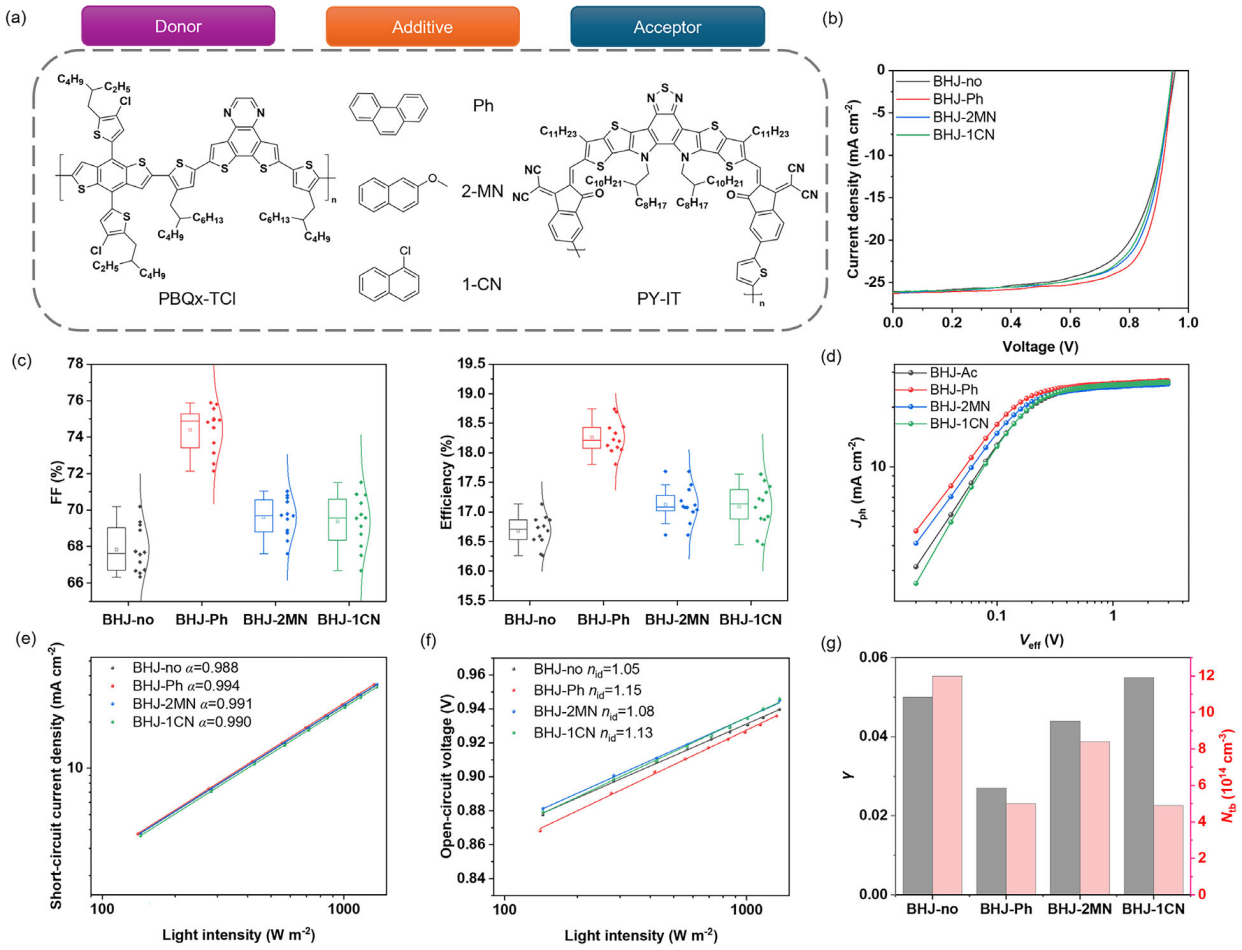
(a) Molecular structure of donor PBQx‐TCl, acceptor PY‐IT, three additives Ph, 2‐MN, 1‐CN. (b) Representative *J–V* curves. (c) Device statistics in FFs and PCEs. (d) *J*_ph_ versus *V*_eff_ measurements. Light‐intensity‐dependent (e) *J*_SC_ and (f) *V*_OC_ measurements. (g) Summary of *γ* (grey) and *N*_tb_ (red) of optimized devices with different additives.

**TABLE 1:** Photovoltaic parameters of PBQx‐TCl:PY‐IT all‐PSCs processed under different conditions.

Group	PCE^a^ [%]	*V*_OC_ ^a^ [V]	FF^a^ [%]	*J*_SC_ ^a^ [mA cm^−2^]
BHJ‐no	17.13 (16.68 ± 0.30)	0.963 (0.956 ± 0.004)	69.4 (67.8 ± 1.3)	25.43 (25.62 ± 0.37)
BHJ‐Ph	18.74 (18.26 ± 0.27)	0.953 (0.943 ± 0.005)	75.9 (74.4 ± 1.3)	26.01 (25.98 ± 0.41)
BHJ‐2MN	17.69 (17.13 ± 0.28)	0.953 (0.946 ± 0.005)	70.7 (69.6 ± 1.1)	26.31 (26.00 ± 0.41)
BHJ‐1CN	17.64 (17.09 ± 0.38)	0.948 (0.947 ± 0.005)	71.5 (69.4 ± 1.5)	25.78 (25.90 ± 0.34)

^a^ Average values with their standard deviations (in parentheses) were obtained from 12 independent devices.

To understand the origin of the FF enhancement, we first evaluated charge generation and collection by measuring the photocurrent density as a function of effective voltage (*V*_eff_) (Figure 1d) [33, 35, 36]. The exciton dissociation efficiency (*η*_diss_ = *J*_ph, SC_ / *J*_sat_) and charge collection efficiency (*η*_coll_ = *J*_ph, MPP_ / *J*_sat_) were extracted (Table S2). BHJ‐Ph exhibits the highest *η*_diss_ (96.95%) and *η*_coll_ (84.04%) among all devices, demonstrating more efficient exciton dissociation and charge collection. Light‐intensity‐dependent measurements further clarify the recombination behavior at short‐circuit and open‐circuit conditions. The dependence of *J*_SC_ on light intensity (*P*) follows a power‐law relationship (*J*_SC_ ∝ *P^α^*). As shown in Figure 1e, *α* values close to unity for all devices further confirm that charge extraction is efficient and that short‐circuit currents are essentially generation limited. The open‐circuit voltage *V*_OC_ as a function of ln(*P*) is plotted in Figure 1f, where the slope equals *n*_id_ *kT*/*q*, with *n*_id_, *k*, *T*, and *q* being the ideality factor, Boltzmann constant, absolute temperature, and elementary charge, respectively. All devices exhibit *n*_id_ values closer to 1 rather than 2, indicating that non‐geminate recombination at open circuit is dominated by bimolecular processes rather than trap‐assisted Shockley‐Read‐Hall recombination [37].

To quantitatively disentangle the recombination channels, we employed capacitance spectroscopy [38, 39] to extract the bias‐dependent charge carrier density and effective mobility (*µ*_eff_), enabling decomposition of recombination current density (*J*_rec_) into bimolecular (*J*_br_) and trap‐assisted (*J*_tb_) components and extraction of *γ* and bulk trap density (*N*_tb_). Fitting details are given in Figure S5 and the **Experimental Methods,** and the resulting parameters are summarized in Figure 1g. BHJ‐no exhibits *γ* of 0.050 and *N*_tb_ of 1.2 × 10^15^ cm^−3^, indicative of both significant bimolecular and trap‐assisted recombination, consistent with its lowest *J*_SC_ and FF. Upon additive incorporation, *N*_tb_ decreases to the 10^14^ cm^−3^ range for all three additives, suggesting that additive engineering effectively reduces trap density in general. However, the evolution of *γ* differs markedly: BHJ‐Ph exhibits the smallest *γ* of 0.027, significantly lower than BHJ‐2MN (*γ* = 0.044) and BHJ‐1CN (*γ* = 0.055).

Compared to high‐performance polymer:NFA systems reported previously [38, 40], all of our devices exhibit *N*_tb_ values of similar order of magnitude but *γ* values roughly one order of magnitude larger, confirming that intrinsically more severe bimolecular recombination is the primary efficiency bottleneck in this all‐PSC system. Encouragingly, Ph nearly halves the *γ* relative to the additive‐free device, whereas 2‐MN and 1‐CN produce only minor or negligible reductions, indicating that Ph is particularly effective at suppressing the bimolecular recombination channel. Transient photovoltage (TPV) (Figure S6a) and charge extraction measurements were performed to verify recombination behavior in the presence of different additives [41, 42, 43]. Under varying illumination intensities at open‐circuit conditions, BHJ‐Ph exhibits the longest carrier lifetime (*τ*) together with the highest extracted carrier density (Figure S6b), further confirming suppressed bimolecular recombination [44]. Importantly, the trend in *γ* closely mirrors the variation in FF and PCE, highlighting *γ* reduction as the key mechanism behind the performance enhancement of BHJ‐Ph.

### 2.2 Molecular Conformation and Packing

To elucidate the microscopic origin of Ph‐assisted *γ* reduction, UV–vis absorption spectroscopy was first used to probe the optical properties of neat PBQx‐TCl (Figure 2a) and PY‐IT (Figure 2b) films processed with different additives. For the donor film without an additive, the 0–1 and 0‐0 vibronic peaks appear at 530 and 559 nm. Upon incorporation of Ph, these peaks red shift to 533 and 569 nm, and the 0‐0/0‐1 intensity ratio increases, indicating enhanced excitonic coupling and backbone order [45]. For 2‐MN, both peaks shift slightly to 531 and 565 nm, while the donor film processed with 1‐CN shows negligible changes. In contrast, Ph and 2‐MN have little effect on the peak positions of PY‐IT. These observations suggest that Ph preferentially modulates the local conformation of PBQx‐TCl over PY‐IT, demonstrating a donor‐selective morphology control. Consistently, normalized UV–vis spectra of blend films (Figure S7) show that Ph induces the largest red shift of donor‐related peaks, confirming similar effects in the blends.

**FIGURE 2:**
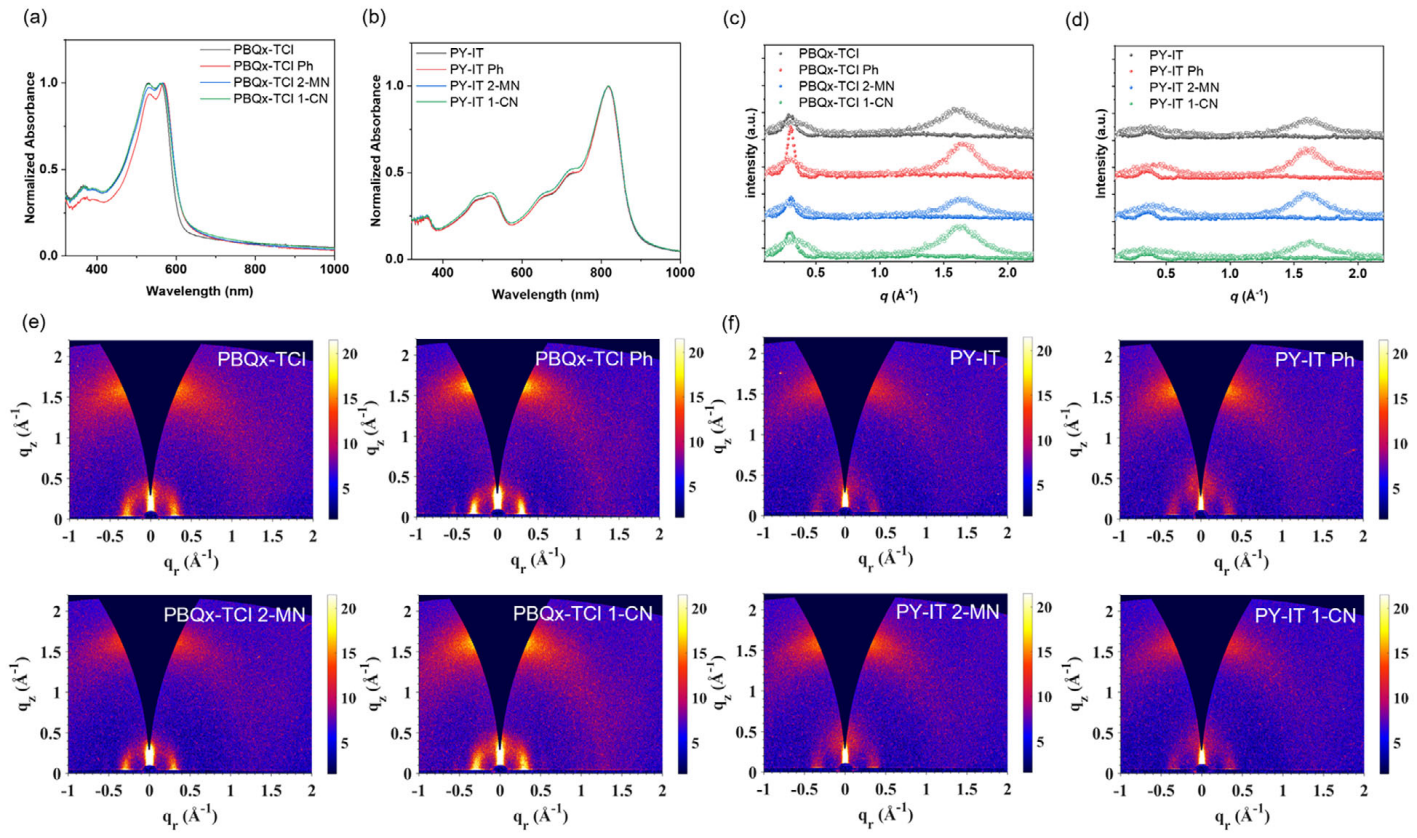
Normalized UV–vis spectra of (a) donor films and (b) acceptor films. GIWAXS linecuts of (c) donor and (d) acceptor (solid dots represent linecuts along the in‐plane direction, while open dots represent linecuts along the out‐of‐plane direction). 2‐D GIWAXS patterns of (e) donor and (f) acceptor with different additives.

Grazing‐incidence wide‐angle scattering (GIWAXS) was then conducted to study how Ph affects crystalline packing. GIWAXS linecuts of donor and acceptor films with different additives are shown in Figure 2c,d, and the corresponding two‐dimensional (2‐D) GIWAXS patterns are presented in Figure 2e,f. For neat PBQx‐TCl without an additive, lamellar (100) and *π–π* peaks (010) at (*q*_r_, *q*_z_) of (0.293, 0) and (0, 1.615) Å^−1^ are observed (Tables S3 and S4), with crystalline coherence lengths [46] (CCLs) of 60 and 16.8 Å, respectively. Among all three additives, Ph has the most pronounced effect in promoting the crystallinity and reducing *d*‐spacings of the donor. With Ph, these peaks shift to (0.302, 0) and (0, 1.650) Å^−1^, corresponding to reduced *d*‐spacings from 21.4 to 20.8 Å and from 3.89 to 3.81 Å, while the CCLs increase to 98 and 21.7 Å. The (002) backbone peak at (0.548, 0) Å^−1^ also becomes more pronounced, signifying substantially enhanced backbone ordering. Together, these observations indicate that Ph strongly promotes the packing order of PBQx‐TCl. In contrast, Ph induces only a modest increase in PY‐IT crystallinity: the *π–π* CCL increases slightly from 18.6 to 20.5 Å, while the *π–π* d‐spacing remains unchanged and the lamellar d‐spacing even increases. These results confirm that Ph exerts a preferential modulation on the donor polymer. GIWAXS patterns of blend films are shown in Figure S8. Due to the similar lattice constants of PBQx‐TCl and PY‐IT, their scattering contributions cannot be fully separated, but BHJ‐Ph exhibits the largest overall *π–π* peak area, indicating enhanced crystallinity beneficial for charge transport.

### 2.3 Surface Morphology and Nanophase Separation

Surface morphology was further investigated using atomic force microscopy (AFM). As shown in Figure 3a, the root‐mean‐square (RMS) roughness of BHJ‐no, BHJ‐Ph, BHJ‐2MN, and BHJ‐1CN is 1.172, 1.051, 1.347, and 1.289 nm, respectively, with BHJ‐Ph exhibiting the relatively smoothest surface, indicative of improved film uniformity. AFM phase images reveal distinct fiber‐like structures for films processed with additives, whereas BHJ‐no shows no apparent features, consistent with grazing‐incidence small‐angle scattering (GISAXS) observations below.

**FIGURE 3:**
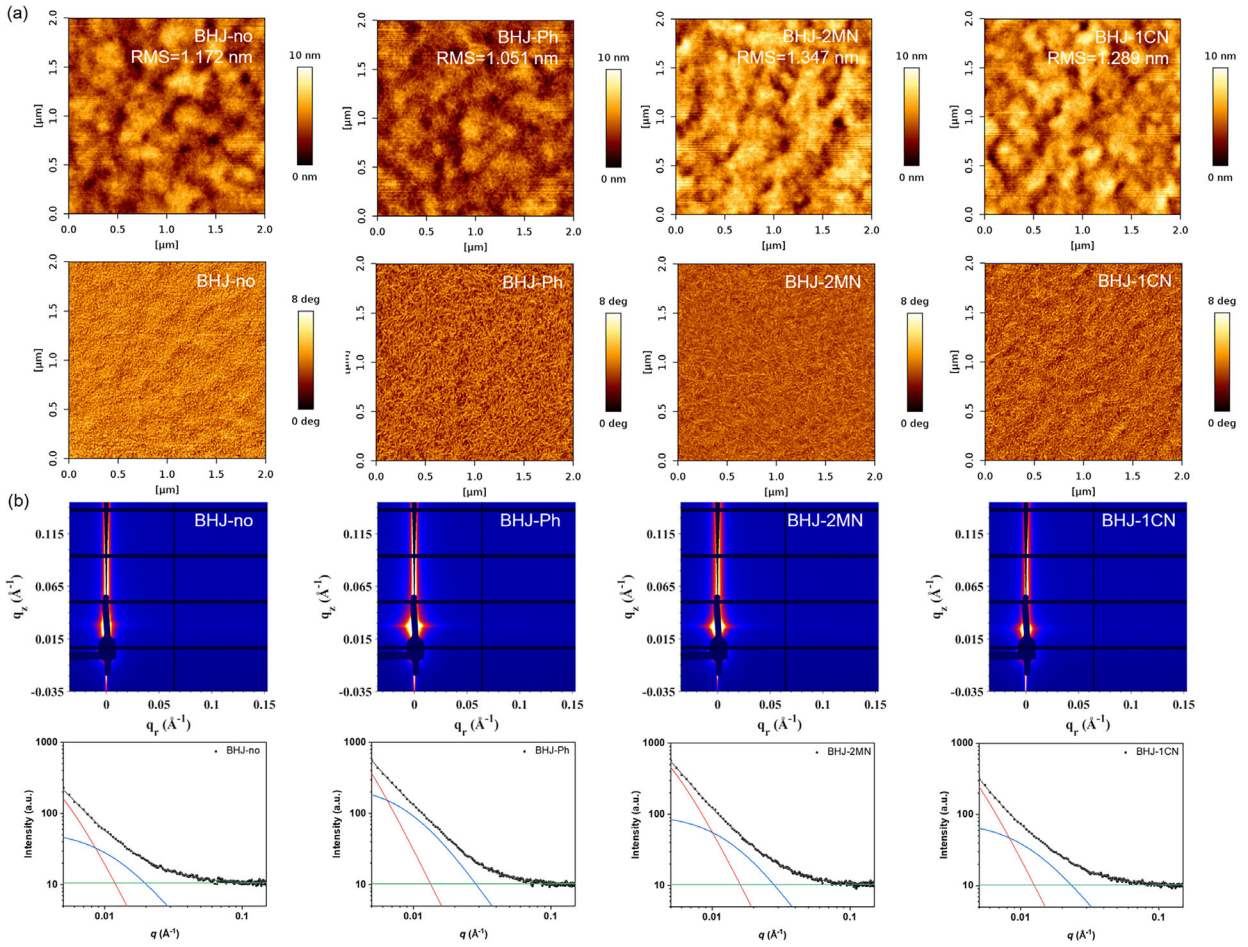
(a) AFM topology and phase images of optimized blend films with different additives. (b) 2‐D GISAXS patterns of blend films with different additives and corresponding linecuts (solid dots). Black solid lines represent the best fitting results; red, blue, and green solid lines represent the contributions from DAB model, fractal model, and background.

GISAXS measurements provide complementary information on bulk nanophase separation [47]. 2‐D GISAXS patterns, corresponding IP linecuts, and fitting results are shown in Figure 3b. The Debye‐Anderson‐Brumberger (DAB) model [48] was applied to quantify the contribution of amorphous D:A intermixed domains, while a fractal network structure factor multiplied by a spherical form factor describes the scattering from crystalline domains [38]. For polymer donor:NFA systems, the crystalline contribution is typically attributed to NFA aggregates [49]; in this all‐polymer system, the similar scattering length densities of PBQx‐TCl and PY‐IT imply low contrast, but analysis of neat film GISAXS (Figure S9) supports attributing the mid‑q crystalline signal to PY‑IT. From the fitting results, we extracted the intermixed domain correlation length (*ξ*), crystalline domain size (2*R*_g_), and the fractal dimension (*D*_f_), summarized in Table 2. All *D*_f_ values lie between 2 and 3, consistent with branched polymeric networks [50]. BHJ‐Ph shows the largest *ξ* (41.3 nm) and 2*R*_g_ (41.7 nm), indicating the enhanced donor‐acceptor phase separation into larger crystalline domains. Both Ph and 2‐MN increase the PY‑IT *D*_f_ to 2.54 and 2.47, respectively, compared to BHJ‐no (2.26) and BHJ‐1CN (2.30). In previous polymer donor:NFA systems, lower *D*_f_ was found to reduce *N*_tb_ by promoting more extended NFA networks; in the present all‐polymer system, where bimolecular rather than trap‐assisted recombination dominates, higher *D*_f_ instead corresponds to less branched, denser acceptor clusters and a reduced donor‐acceptor interfacial area, which further lowers the carrier encounter probability. In the ternary system (PBQx‐TCl:PY‐IT:PY‐DT‐X) with Ph, the *D*_f_ value further increases to 2.65 (Figure S10 and Table S7), corresponding well with the improved FF and PCE. This highlights that the optimal morphology descriptor (e.g., *D*_f_) depends sensitively on the dominant recombination mechanism.

**TABLE 2:** Fitting parameters of optimized blend films without or with different additives.

Additive	*ξ* [nm]	*D*_f_	2*R*_g_ [nm]
BHJ‐no	26.1	2.26	34.1
BHJ‐Ph	41.3	2.54	41.7
BHJ‐2MN	25.5	2.47	29.5
BHJ‐1CN	33.7	2.30	34.1

### 2.4 Evaporation Kinetics and Simulations

To rationalize the formation mechanism of distinct morphology, we next investigated how the additives regulate film formation kinetics during solvent and additive evaporation. All three fused‑aromatic additives (Ph, 2‑MN and 1‑CN) are readily soluble in chloroform, but they differ strongly in volatility: Ph is a crystalline solid (melting point ∼100°C) that starts to sublime when approaching its melting range [30, 51, 52], enabling a mild evaporation during annealing; 2‑MN is a low‑melting (∼72°C [13]) solid that begins to volatilize in the 40°C–90°C range, leaving the film more rapidly than Ph; 1‑CN is a high‑boiling liquid (∼260°C [53]) that remains largely in the film even after annealing [27, 54, 55]. Thermogravimetric analysis (TGA) was performed (Figure S11a) to verify the sample mass‐loss behavior [56] of 2‐MN and Ph. Ph shows a higher *T*_5%_ (temperature corresponding to 5% weight loss) of 175°C compared to 133°C for 2‐MN. For spin‐coated additive films [57], Ph and 2‐MN will be removed within 1 min without residual, which further verifies the good annealing volatility of the two solid additives (Figure S11b).

These volatility differences are directly reflected in film formation kinetics as revealed by in situ UV–vis absorption. Owing to the use of low‐boiling chloroform and the temporal resolution of our setup, all films show a similar drying time of ∼ 1 s during spin‐coating (Figure S12). During the subsequent thermal annealing at 90°C, BHJ‐no (Figure 4a) exhibits almost unchanged absorption peak positions, indicating limited modulation of molecular packing. For BHJ‐1CN (Figure 4b), peak positions also remain nearly unchanged, consistent with the high boiling point of 1‐CN, which hampers its removal and leaves little room for further morphology evolution. By contrast, BHJ‐2MN (Figure 4c) and BHJ‐Ph (Figure 4d) show pronounced spectral evolution during annealing. The duration of peak shifts exceeds 60 s for BHJ‐Ph, much longer than the ∼15 s observed for BHJ‐2MN, reflecting their different volatility and highlighting the substantially longer morphology evolution window with Ph.

**FIGURE 4:**
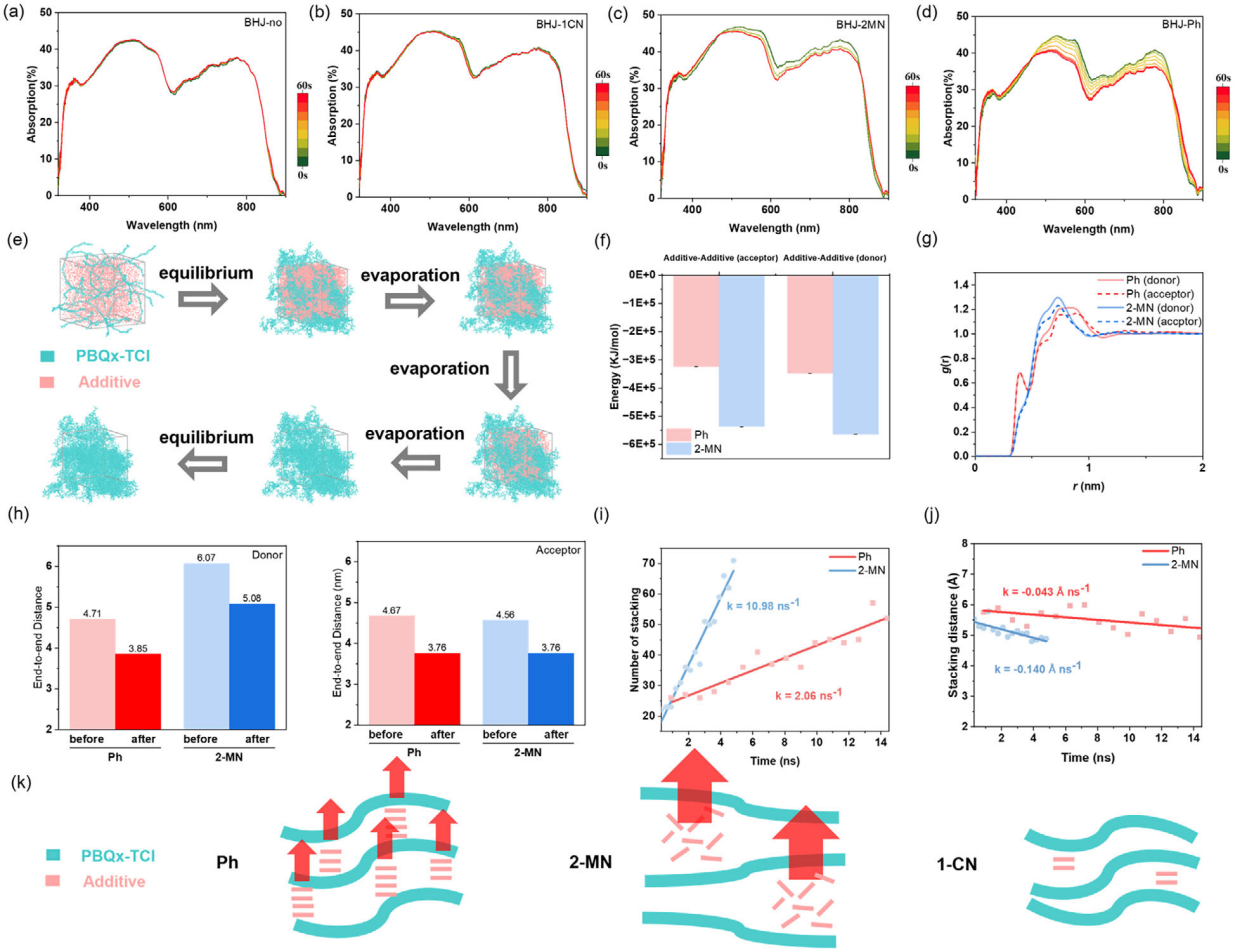
In situ UV absorption of (a) BHJ‐no; (b) BHJ‐1CN; (c) BHJ‐2MN; (d) BHJ‐Ph during the thermal annealing process. (e) Schematic of AA‐MD simulation process. (f) Calculated interaction energy between solid additive molecules. (g) radial distribution function g(r) of solid additives in donor films (solid lines) and in acceptor films (dashed lines). (h) End‐to‐end distance of donor and acceptor molecules with different additives before and after thermal annealing. (i) Change of packing numbers and (j) packing distance during the evaporation process of donor films with Ph and 2‐MN. (k) Schematic of additive distribution in donor films.

All‐atom molecular‐dynamics (AA‐MD) simulations were employed to simulate the evaporation process and self‐aggregation of Ph and 2‐MN to further understand their distinct morphology control capabilities (Figure 4e, donor as an example, details are included in the **Experiment Methods**). Van der Waals interactions are found to dominate. 2‐MN exhibits a significantly stronger tendency to self‐aggregate, with additive‐additive interaction energy almost twice that of Ph (Figure 4f), consistent with previous reports [13, 19]. Radial distribution function [58, 59, 60] (*g*(*r*), Figure 4g) reveals that 2‐MN does not exhibit significant packing within 5 Å, whereas Ph shows a distinct peak at ∼4 Å, suggesting that the three‐ring structure of Ph promotes stronger *π–π* interaction itself.

End‐to‐end distance (*l*) distributions [61, 62] of donors and acceptors before and after additive evaporation were also simulated (Figure 4h). Ph‐assisted donor film exhibits more compact donor configuration before and after Ph evaporation (from 4.71 to 3.85 Å) compared with 2‐MN‐assisted film (from 6.07 to 5.08 Å). In contrast, Ph acts as a more efficient spacer in acceptor films with end‐to‐end distances of 4.67 Å compared to 2‐MN of 4.56 Å. The end‐to‐end distances become similar after evaporation, confirming the donor‐selective morphology control of both additives. The donor packing evolution rates in stacking numbers (Figure 4i) and distances (Figure 4j) both suggest slower packing in the presence of Ph, in agreement with in situ UV results. As illustrated in Figure 4k, the steady evaporation of uniformly distributed Ph between donor molecules, together with a more efficient spacer effect of Ph between acceptor molecules as mentioned before, cooperatively provides longer evolution time and larger and uniform relaxation volume for the acceptor domain to grow into larger and denser domains with increased *D*_f_ as observed in GISAXS.

## 3 Conclusions

In conclusion, we demonstrate that bimolecular recombination constitutes the dominant efficiency bottleneck in high‐performance all‐PSCs and that evaporation kinetics engineering provides an effective strategy for morphology control. By introducing phenanthrene as a volatile solid additive for the first time in all‐PSCs, we show that its moderate volatility and weak self‐aggregation tendency enable donor‐selective crystallization while providing sufficient relaxation time and space for the growth of acceptor domains with increased fractal dimension. This cooperative structural evolution reduces excessive donor‐acceptor interfacial area and suppresses bimolecular recombination, nearly halving the Langevin reduction factor (*γ*), thereby leading to substantial enhancement in FFs. As a result, PBQx‐TCl:PY‐IT‐based binary and ternary all‐PSCs achieve a PCE of 18.74% and 19.35%, respectively, demonstrating the generality of this approach. Overall, this work establishes rational selection rules for volatile solid additives, emphasizing the importance of balancing molecular interaction, self‐aggregation tendency, and evaporation kinetics. More broadly, it provides a general framework for controlling multiscale morphology to suppress bimolecular recombination, offering new opportunities to further advance the efficiency of all‐PSCs.

## Author Contributions

X.L. conceived the idea and supervised this work. D.F., L.M., and C.L. contributed equally to this work. D.F. performed all‐PSC device fabrication and characterization. L.M. and X.C. conducted the AA‐MD simulations. C.L. and H.Y. synthesized PY‐DT‐X. Y.F. and L.X. performed capacitance spectroscopy experiments and the corresponding analysis. H.L. and D.F. performed AFM and GIWAXS measurements. P.F.C, C.‐J.S., U.‐S.J., and D.F. performed GISAXS measurements. Z.Y. and J.W. performed the transient optoelectronic characterization. Y.W. and L.Z. supported device optimization. P.W.‐K.F and G.L. conducted in situ UV–vis absorption. D.F. and X.L. prepared the manuscript. All authors provided revisions.

## Conflicts of Interest

The authors declare no conflicts of interest.

## Supporting information




**Supporting File**: advs77114‐sup‐0001‐SuppMat.docx.

## Data Availability

The data that support the findings of this study are available from the corresponding author upon reasonable request.
