# Supplementary material for: Evaporation Kinetics Engineering Suppressed Bimolecular Recombination in All‐Polymer Solar Cells
Source: Adv Sci (Weinh). 2026 Aug 17;13(61):e77114. doi: 10.1002/advs.77114 (PMC13479588; doi:10.1002/advs.77114)
Supplement: Supplementary file 1 [file ADVS-13-e77114-s001.docx]

**Evaporation Kinetics Engineering Suppressed Bimolecular Recombination in All-polymer Solar Cells**

Dinglong FENG^a^, Le MEI^b^, Chao LI^c,d^, Yuang FU^a^, Heng LIU^a^, Luhang XU^a^, Pok Fung CHAN^a^, Zhixiang YANG^e^, Yiming WANG^f^, Chun-Jen Su^g^, U-Ser Jeng^g,h^, Patrick Wai-Keung FONG^i^, Lijian ZUO^f^, Gang LI^j^, He YAN^c^, Jiaying WU^e^, Xian-Kai CHEN^b^, Xinhui LU^a*^

a. Department of Physics, The Chinese University of Hong Kong, New Territories, Hong Kong, 999077, China.

b. Institute of Functional Nano & Soft Materials (FUNSOM), Joint International Research Laboratory of Carbon-Based Functional Materials and Devices, State Key Laboratory of Bioinspired Interfacial Materials Science, Soochow University, Suzhou 215123, P. R. China.

c. Department of Chemistry and Hong Kong Branch of Chinese National Engineering Research Center for Tissue Restoration and Reconstruction, Hong Kong University of Science and Technology, Kowloon, Hong Kong 999077, P. R. China.

d. Hangzhou International Innovation Institute, Beihang University, Hangzhou 311115, P. R. China.

e. Advanced Materials Thrust, Function Hub, The Hong Kong University of Science and Technology (Guangzhou), Nansha, Guangzhou, 511453 P. R. China.

f. State Key Laboratory of Silicon Materials; MOE Key Laboratory of Macromolecular Synthesis and Functionalization, Department of Polymer Science and Engineering, Zhejiang University, Hangzhou 310027, P. R. China.

g. National Synchrotron Radiation Research Centre, Hsinchu Science Park, Hsinchu 30092, Taiwan.

h. Department of Chemical Engineering & College of Semiconductor Research, National Tsing Hua University, Hsinchu 300044, Taiwan.

i. Department of Electrical and Electronic Engineering, The Hong Kong Polytechnic University, Hung Hom, Kowloon, Hong Kong 999077, China.

j. Department of Electrical and Electronic Engineering, Research Institute of Smart Energy (RISE), Photonic Research Institute (PRI), The Hong Kong Polytechnic University, Hung Hom, Kowloon, Hong Kong 999077, China.

^✉^ email: [xinhui.lu@cuhk.edu.hk](mailto:xinhui.lu@cuhk.edu.hk)

## Experiment Method

**Materials:** Chloroform (99.5%), methanol (99.8%), Zn(CH_3_COO)_2_·2H_2_O, 2-Methoyethanol, ethanolamine, MoO_3_ powder, and 1-chloronapthalene (1-CN) (96%) were purchased from Sigma-Aldrich. Absolute ethanol (HPLC) was purchased from Scharlab S.L. 2-methoxynaphthalene (2-MN) (98%), phenanthrene (Ph) (99%), and 2PACz (98%) were purchased from Meryer. PBQx-TCl and PY-IT were purchased from Solarmer Inc (Beijing). PY-DT-X was synthesized by Chao LI. PNDIT-F3N was purchased from eFlexPV Ltd. PEDOT:PSS (Clevios P VP AI 4083) was purchased from Heraeus Ltd. All solvents and reagents were used as received without further purification.

**Device Fabrication and Characterizations**

All devices were fabricated based on a conventional device structure of ITO/2PACz/active layer/PNDIT-F3N/Ag. The patterned ITO glass substrates were subsequently cleaned in detergent, deionized water, acetone, and isopropanol for 15 minutes twice for each. The cleaned substrates were dried at 100℃ and pretreated by UV-ozone for 20 minutes before use. The substrates were then transferred into a glove box filled with N_2_. A self-assembly interlayer was fabricated by spin-coating 0.2 mg ml^-1^ solution of 2PACz in ethanol at 4000 rpm for 30 s without further rinsing and then thermal annealed at 100 ℃ for 10 min. PBQx-TCl and PYIT were dissolved at a mass ratio of 1:1.2 with a donor concentration of 5 mg ml^-1^ chloroform combined with different additives. The optimized concentrations of different additives were 7 mg ml^-1^ for Ph and 2-MN, and 0.2 vol% for 1-CN. For ternary devices, PBQx-TCl, PYIT, and PY-DT-X were dissolved at a mass ratio of 1:1:0.3 with a donor concentration of 5 mg ml^-1^. The blend solution was stirred and kept at 40 ºC for 1 hour before spin-coating. The optimized thickness of active layer was controlled to 90 nm, and all devices underwent thermal annealing at 90 ºC for 5min. PNDIT-F3N (0.5 mg ml^-1^ in methanol, with 0.5 vol% ethanoic acid) was spin-coated on the top of the active layer at 2000 rpm for 30 s, followed by thermal evaporation of Ag electrode (about 100 nm) at < 10^-4^ Pa. For AM 1.5G light-soaking test, solution-processed ZnO was used to fabricate inverted-structure devices. The ZnO precursor was prepared by mixing Zn(CH_3_COO)_2_·2H_2_O (100 mg) and ethanolamine (28.29 μL) in 973 μL 2-Methoyethanol. The ZnO was then deposited on ITO glass by spin-coating at 4000 rpm for 30 s followed by thermal annealing on the hotplate at 200 ^o^C for 30 min. 5nm MoO_3_ was thermally evaporated as an electron-transporting layer. For ambient storage tests, PEDOT:PSS was used as a hole-transporting layer by spin-coating at 4000 rpm for 30s followed by thermal annealing on the hotplate at 120 ^o^C for 20 min. All other steps were controlled to be the same.

The current density-voltage (*J*-*V*) curves of devices were measured using Keithley 2400 Source Meter ( -0.20 V to 1.20 V, 0.01 V per step, forward scan) in N_2_ filled glove box under AM 1.5G (100 mW cm^-2^) conditions using a solar simulator (SS-F55-3A, Enlitech). The light intensity was calibrated by a reference silicon solar cell (SRC2020, Enlitech). The device area was determined by the overlap between the top and bottom electrodes which was 0.0439 cm^2^, as confirmed by the optical microscope. The external efficiency (EQE) was measured by the QE-R quantum efficiency system (QE-R3011, Enlitech) in a wavelength range of 300-1000 nm, 5 nm per step. For *J*_ph_-*V*_eff_ measurements, dark and light currents (*J*_light_ and *J*_dark_) were measured under the range of -2 V to 2 V, 0.05 V per step, so that *J*_ph_ can be derived as *J*_ph_ = *J*_dark_ – *J*_light_. The bias applied to the active layer, *V*_cor_, can be obtained from the equation *V*_cor_ = *V* - *J*_light_ × *R*_s_ where *R_s_* is the series resistance of the device derived from the differential resistance of the dark *J-V* curve under the forward bias of 2 V.

**Optical Characterizations**

The absorption spectra of thin films were measured using PerkinElmer lambda 950UV/Vis/IR with wavelength scanned from 1000 nm to 320 nm, 1 nm per step, using the 2-D model. Samples were deposited on quartz substrates.

**Voltage-dependent Capacitance Spectroscopy**

All devices were encapsulated and tested in air. A 10 mV AC bias was applied to the device with frequency scanning from 4 MHz to 10 kHz, and the corresponding AC current was measured to obtain the complex impedance of the device as a function of frequency. The total capacitance (*C*_cor_) of the device was derived from the complex impedance using **Equation 1**, excluding the effect of series resistance (*R*_s_) and parasitic inductance (*L*_I_).^[1-2]^

$$\begin{aligned} C_{cor}=-\frac{1}{\omega}\left[ \frac{Z^{''}-\omega L_{I}}{\left( Z^{'}-R_{s} \right)^{2}+\left( Z^{''}-\omega L_{I} \right)^{2}} \right]\#(1) \end{aligned}$$

Where Z’ and Z’’ are the real and imaginary parts of the complex impedance, and *ω* is the angular frequency. After subtracting the *C*_cor_ by the geometric capacitance (*C*_g_, measured under the dark at -1 V), the total capacitance associated with the active layer only (*C*_T_) was obtained, and the chemical capacitance (*C*_μ_) was derived from the saturated *C*_T_ at low frequency. Measurements were carried out with devices under 1 sun-equivalent LED illumination and constant background bias (*V*_DC_) scanning from -1 V to 1 V. Charge carrier density within the active layer was obtained by integrating the *C*_u_ with respect to the corrected applied bias (*V*_cor_=*V*_app_-*JR*_s_) using **Equations 2 and 3**

$$\begin{aligned} n\left( V_{cor} \right)=n_{sat}+\frac{1}{qAL}\int_{V_{sat}}^{V_{cor}} C_{\mu}dV_{cor}\#(2) \end{aligned}$$

$$\begin{aligned} n_{sat}=\frac{1}{qAL}C_{sat}\left( V_{0}-V_{sat} \right)\#\left( 3 \right) \end{aligned}$$

where *C*_sat_ is the saturated *C*_μ_ measured at *V*_sat_ (-1 V) under 1 sun. V_0_ is forward bias at which *J*_ph_ equals zero. *L* is the thickness of the active layer (around 100 nm). Effective charge carrier mobilities (*μ*_eff_) at various *V*_cor_ were obtained via Equation 4.^[3]^

$$\begin{aligned} \mu_{eff}\left( n,V_{cor} \right)=\frac{J\left( V_{cor} \right)\cdot L}{2qn\left( V_{cor} \right)\cdot\left[ V_{cor}-V_{OC} \right]}\#\left( 4 \right) \end{aligned}$$

*J*_rec_ equals the difference between *J*_ph_ and *J*_sat_. We then fitted *J*_rec_ as a function of *V*_cor_ using the linear superposition of bimolecular (*J*_br_) and bulk trap-assisted recombination (*J*_tb_) currents, as shown in **Equations 5 and 6**

$$\begin{aligned} J_{br}=qLk_{bm}n^{2}=\frac{{2q}^{2}L}{\varepsilon_{0}\varepsilon_{r}}\gamma\mu_{eff}n^{2}\#\left( 5 \right) \end{aligned}$$

$$\begin{aligned} J_{tb}=qLk_{tb}n=\frac{q^{2}L}{\varepsilon_{0}\varepsilon_{r}}\mu_{eff}N_{tb}n\#\left( 6 \right) \end{aligned}$$

where *q* is the elementary charge, *ε*_0_ and *ε*_r_ are the permittivity of free space and dielectric constant. The Langevin reduction factor *γ* for bimolecular recombination and bulk trap density *N*_tb_ were left as fitting parameters. All curves could be fitted without involving surface recombination current, which is reasonable considering that both charge transport layers used have been shown to exhibit good carrier selectivity for the PBQx-TCl:PY-IT system.

**Transient Optoelectronic Characterizations**

Transient photovoltage (TPV) measurements and charge extraction (CE) at open circuit were measured using an all-in-one characterization system (PAIOS, Fluxim, Switzerland).

**Thermogravimetric Analysis (TGA)**

TGA was performed on a Perkin-Elmer Pyris 6 TGA equipment under a nitrogen flow. The temperature increasing rate was set at the same rate of 10 °C min^-1^.

**AFM Measurements**

JPK NanoWizard NanoOptics atomic force microscope (AFM) with a Multi-75G tip (Budget Sensors) was used to obtain topology and phase images of thin films. Measurements were performed using tapping mode with the tip oscillating at a fixed frequency (~75 kHz), and an amplitude above the sample surface. 2 × 2 um images with 512 × 512 pixels were obtained for each sample. The root-mean-square (RMS) fluctuations in height were obtained using the JPKSPM Data processing software package.

**GIWAXS, GISAXS Measurements and Fitting**s: GIWAXS measurements for neat and blend films were carried out with a Xeuss 2.0 SAXS/WAXS laboratory beamline using a Cu X-Ray source (8.05 keV, 1.54 Å) and a Pilatus 3R 300K detector. The incidence angles were 0.2°. GISAXS measurements for blend films were conducted at 23A SWAXS beamline at the National Synchrotron Radiation Research Center (NSRRC), Hsinchu, Taiwan, using a 10 keV primary beam, 0.15° incident angle. All samples were spin-coated on the UV-ozone-treated silicon substrates.

The GISAXS profiles were fitted using the general **Equation 7**:

$$\begin{aligned} I\left( q \right)=\frac{A_{1}}{\left[ 1+\left( q\cdot\xi\right)^{2} \right]^{2}}+A_{2}\left\langle P\left( q,R_{gc} \right) \right\rangle S_{frac}\left( q,R_{gc},\eta,D_{f} \right)+B\#\left( 7 \right) \end{aligned}$$

where *q* is the scattering vector. The amorphous intermixed region was fitted using the Debye-Anderson-Brumberger (DAB) model (the 1^st^ term) with perfector *A*_1_ and intermixed domain correlation length *ξ*. The crystalline domains were fitted using spherical form factor *P*(*q*, *R*_gc_) multiplied by fractal structure factor (the 2^nd^), with domain size represented by twice the radius of gyration 2*R*_g_.^[4]^ The 3^rd^ term represents the incoherent background scattering. All blend films fit this model well.

**In-situ UV-vis Absorption**

The in-situ UV-vis spectra were measured using an F20 spectrometer (Filmetrics, Inc.). The light source was a halogen lamp with a spot size of 1.5 mm. 400 nm Ag was coated on the backside of the patterned ITO glass to increase the reflection. The spin-coating and thermal annealing process was controlled to be the same as the device fabrication condition. The time resolution is around 0.3 s.

**All-atom Molecular-dynamics Simulations (AA-MD)**

All the AA-MD simulations were carried out using the GROMACS2021 software suite, equipped with the General Amber Force Field (GAFF).^[5-6]^ The atom charges were fitted using the Restrained Electrostatic Potential (RESP) method, employing the B3LYP functional and the 6-31 basis set. By employing a generalized solvent evaporation method, we closely mirrored the experimental conditions, ensuring the removal of solvents and additives at predetermined intervals.

For the neat films of PBQx-TCl, the initial models were built by randomly inserting additives and the PBQx-TCl polymer chain. Each box contained 25 PBQx-TCl polymer chains with 20 repeating units. For the neat films of PY-IT, each box contained 46 PY-IT polymer chains with 10 repeating units to simulate the oligomer property and control the weight ratio to approximately 1:1.2. Additionally, the additives were also controlled to the same weight ratio as the real experiment condition (5449 molecules for Ph, 6138 molecules for 2-MN, and 2037 molecules for 1-CN in each cells). To simulate the experimental annealing process, all cells were first equilibrated at 300 K for 30 ns, 363 K for 30 ns, and then annealed to 300 K for 30 ns. Since 1-CN is widely reported to remain in the film, we consider this state as the final result both before and after evaporation. For Ph and 2-MN, we set different evaporation rates, Ph 100 molecules/300 ps and 2-MN 400 molecules/300 ps, according to the different evaporation times. After the evaporation, the cells were equilibrated at 300 K for 30 ns, 363 K for 30 ns, and annealed to 300 K for 30 ns, the whole simulation process is 210 ns.

During the simulations, the LINCS algorithm was applied to constrain the covalent bonds involving hydrogen atoms.^[7]^ The time step of the simulations was 1.0 fs. All the simulations were performed in periodic boundary conditions. The pressure and the temperature were controlled with the Parrinello-Rahman barostat coupled at 1 atm and the V-rescale thermostat, respectively.^[8-9]^ The nonbonded interactions were truncated at a cut-off distance of 12 Å, while the particle mesh Ewald (PME) method was used to calculate the long-range electrostatic interactions.^[10]^ The graphics and visualization analyses were processed by the Visual Molecular Dynamics (VMD) program.^[11]^

**Statistical Analysis**

In **Figure 1c** and **Figure S1**, box plots and scatter plots, together with a normal distribution curve on the right, were used to show the variance of device parameters of 12 individual devices. For the stability test in **Figure S3**, the curves represent the average value from 6 individual devices. The error bar represents the standard deviation.

**Figure S1.** a) Device statistics in *V*_OC_ and b) *J*_SC_.

**Figure S2.** a) External quantum efficiency measurement of BHJ-no; b) BHJ-Ph and corresponding integral *J*_SC_.

**Figure S3.** a) Thermal stability of devices with different additives based on ITO/2PACz/Active layer/PNDIT-F3N/Ag structure under 60°C stress. Each group contains 6 individual devices. b) AM 1.5G light-soaking test of devices with different additives based on the ITO/ZnO/Active layer/MoO_3_/Ag structure. Each group contains 6 individual devices. c) Ambient storage test based on ITO/PEDOT:PSS/Active layer/PNDIT-F3N/Ag after encapsulation under room temperature, 60% relative humidity. Each group contains 6 individual devices.

**Figure S4.** External quantum efficiency measurement of PBQx-TCl:PY-IT:PY-DT-X ternary system.

**Figure S5.** a) Fitting results (*J*_br_ and *J*_tb_) of BHJ-no; b) BHJ-Ph; c) BHJ-2MN; d) BHJ-1CN.

**Figure S6.** a) Representative TPV decay curves (dots) under 10% intensity illumination and corresponding carrier lifetime extracted from single exponential decay fitting (solid lines). b) TPV lifetime versus carrier density plot obtained from different illuminations.

**Figure S7.** Normalized UV-Vis spectra of blend films.

**Figure S8.** 2-D GIWAXS patterns of blend films with different additives and corresponding linecuts.

**Figure S9.** a) 2-D GISAXS pattern of PBQx-TCl; b) 2-D GISAXS pattern of PY-IT; c) GISAXS linecuts of PBQx-TCl and PY-IT.

**Figure S10.** a) 2-D GISAXS pattern of PBQx-TCl:PY-IT:PY-DT-X ternary system; b) GISAXS linecuts of the ternary system.

**Figure S11.** a) TGA curves for three additives. b) Volatilization behaviors of Ph and 2-MN in thin film states.

**Figure S12.** In-situ 2-D UV-vis absorption of a) BHJ-no; b) BHJ-Ph; c) BHJ-2MN; d) BHJ-1CN during the spin-coating process.

**Table S1.** Photovoltaic parameters of PBQx-TCl:PY-IT:PY-DT-X ternary system with mass ratio of 1:1:0.3.

| Group | *V*_OC_^a^ [V] | PCE^a^ [%] | FF^a^ [%] | *J*_SC_^a^ [mA cm^-2^] |
| --- | --- | --- | --- | --- |
| Ternary | 0.945  (0.944 ± 0.005) | 19.35  (18.71 ± 0.38) | 76.7  (75.1 ± 0.86) | 26.79  (26.41 ± 0.51) |

^a^ Average values with their standard deviations (in parentheses) were obtained from 12 independent devices.

**Table S2.** Photocurrent density, charge dissociation, and collection efficiencies of the optimized device with different additives.

| Group | *J*_ph1_ | *J*_ph2_ | *J*_sat_ | *η*_diss_ | *η*_coll_ |
| --- | --- | --- | --- | --- | --- |
| BHJ-no | 25.85 | 21.63 | 26.80 | 96.46% | 80.74% |
| BHJ-Ph | 26.36 | 22.85 | 27.19 | 96.95% | 84.04% |
| BHJ-2MN | 25.36 | 21.91 | 26.22 | 96.72% | 83.54% |
| BHJ-1CN | 25.89 | 21.93 | 26.79 | 96.65% | 81.87% |

**Table S3.** Summary of calculated results of neat donor and acceptor films along the IP direction.

| IP | *q* [Å^-1^] | *d* [Å] | Peak Area [a.u.] | CCL [Å] |
| --- | --- | --- | --- | --- |
| PBQx-TCl (100) peak | 0.293±0.005 | 21.4±0.4 | 0.74±0.03 | 60±1 |
| PBQx-TCl Ph  (100) peak | 0.302±0.005 | 20.8±0.4 | 1.12±0.03 | 98±4 |
| PBQx-TCl Ph (002) peak | 0.548±0.005 | 11.5±0.1 | 0.08±0.01 | N/A |
| PBQx-TCl 2-MN (100) peak | 0.300±0.005 | 20.9±0.3 | 0.58±0.02 | 74±3 |
| PBQx-TCl 1-CN (100) peak | 0.298±0.005 | 21.1±0.4 | 0.86±0.03 | 63±1 |
| PY-IT (100) peak | 0.357±0.007 | 16.8±0.3 | 0.25±0.02 | 50±4 |
| PY-IT Ph  (100) peak | 0.347±0.006 | 18.1±0.3 | 0.36±0.02 | 48±2 |
| PY-IT 2-MN  (100) peak | 0.356±0.006 | 17.6±0.3 | 0.33±0.02 | 49±3 |
| PY-IT 1-CN  (100) peak | 0.351±0.006 | 17.9±0.3 | 0.36±0.03 | 39±2 |

**Table S4.** Summary of calculated results of neat donor and acceptor films along the OOP direction.

| OOP | *q* [Å^-1^] | *d* [Å] | Peak Area [a.u.] | CCL [Å] |
| --- | --- | --- | --- | --- |
| PBQx-TCl (010) peak | 1.615±0.008 | 3.89±0.02 | 2.88±0.06 | 16.8±0.3 |
| PBQx-TCl Ph  (010) peak | 1.650±0.007 | 3.81±0.02 | 3.06±0.04 | 21.7±0.4 |
| PBQx-TCl 2-MN (010) peak | 1.646±0.007 | 3.82±0.02 | 1.68±0.04 | 20.7±0.5 |
| PBQx-TCl 1-CN (010) peak | 1.630±0.007 | 3.85±0.02 | 3.48±0.06 | 17.8±0.3 |
| PY-IT  (010) peak | 1.614±0.008 | 3.89±0.02 | 1.64±0.05 | 18.6±0.4 |
| PY-IT Ph (010) peak | 1.618±0.007 | 3.88±0.02 | 2.60±0.05 | 20.5±0.3 |
| PY-IT 2-MN (010) peak | 1.612±0.006 | 3.90±0.01 | 2.29±0.04 | 20.4±0.3 |
| PY-IT 1-CN (010) peak | 1.629±0.009 | 3.85±0.02 | 1.63±0.05 | 18.4±0.4 |

**Table S5.** Summary of calculated results of IP linecuts of blend films with different additives.

| IP | *q* [Å^-1^] | *d* [Å] | Peak Area [a.u.] | CCL [Å] |
| --- | --- | --- | --- | --- |
| BHJ-no | 0.313±0.004 | 20.10±0.04 | 1.05±0.02 | 42.2±0.1 |
| BHJ-Ph | 0.316±0.004 | 19.86±0.04 | 1.40±0.03 | 45.2±0.1 |
| BHJ-2MN | 0.315±0.004 | 19.92±0.04 | 1.18±0.02 | 45.5±0.2 |
| BHJ-1CN | 0.316±0.004 | 19.86±0.04 | 1.31±0.02 | 48.3±0.1 |

**Table S6.** Summary of calculated results of OOP linecuts of blend films with different additives.

| OOP | *q* [Å^-1^] | *d* [Å] | Peak Area [a.u.] | CCL [Å] |
| --- | --- | --- | --- | --- |
| BHJ-no | 1.652±0.006 | 3.80±0.01 | 3.84±0.06 | 18.06±0.03 |
| BHJ-Ph | 1.657±0.005 | 3.79±0.01 | 5.44±0.08 | 19.33±0.04 |
| BHJ-2MN | 1.658±0.005 | 3.79±0.01 | 4.48±0.07 | 18.81±0.04 |
| BHJ-1CN | 1.647±0.005 | 3.82±0.01 | 5.22±0.07 | 19.04±0.04 |

**Table S7.** Fitting parameters of the ternary device.

| Group | *ξ* [nm] | *D*_f_ | 2*R*_g_ [nm] |
| --- | --- | --- | --- |
| Ternary | 42.7 | 2.65 | 36.7 |

**Reference**

[1]	Brus, V. V., Proctor, C. M., Ran, N. A., Nguyen, T. Q., "Capacitance Spectroscopy for Quantifying Recombination Losses in Nonfullerene Small‐Molecule Bulk Heterojunction Solar Cells," *Advanced Energy Materials* 6, no. 11 (2016), <https://doi.org/10.1002/aenm.201502250>.

[2]	Brus, V. V., "On impedance spectroscopy analysis of nonideal heterojunctions," *Semiconductor Science and Technology* 27, no. 3 (2012), <https://doi.org/10.1088/0268-1242/27/3/035024>.

[3]	Vollbrecht, J., Brus, V. V., Ko, S. J., et al., "Quantifying the Nongeminate Recombination Dynamics in Nonfullerene Bulk Heterojunction Organic Solar Cells," *Advanced Energy Materials* 9, no. 32 (2019), <https://doi.org/10.1002/aenm.201901438>.

[4]	Liao, H. C., Tsao, C. S., Lin, T. H., et al., "Quantitative nanoorganized structural evolution for a high efficiency bulk heterojunction polymer solar cell," *J Am Chem Soc* 133, no. 33 (2011), <https://doi.org/10.1021/ja202977r>.

[5]	Wang, J., Wolf, R. M., Caldwell, J. W., Kollman, P. A., Case, D. A., "Development and testing of a general amber force field," *J Comput Chem* 25, no. 9 (2004), <https://doi.org/10.1002/jcc.20035>.

[6]	Abraham, M. J., Murtola, T., Schulz, R., et al., "GROMACS: High performance molecular simulations through multi-level parallelism from laptops to supercomputers," *SoftwareX* 1-2(2015), <https://doi.org/10.1016/j.softx.2015.06.001>.

[7]	Hess, B., Bekker, H., Berendsen, H. J. C., Fraaije, J. G. E. M., "LINCS: A linear constraint solver for molecular simulations," *Journal of Computational Chemistry* 18, no. 12 (1997), <https://doi.org/10.1002/(sici)1096-987x(199709)18:12><1463::Aid-jcc4>3.0.Co;2-h.

[8]	Parrinello, M., Rahman, A., "Polymorphic transitions in single crystals: A new molecular dynamics method," *Journal of Applied Physics* 52, no. 12 (1981), <https://doi.org/10.1063/1.328693>.

[9]	Bussi, G., Donadio, D., Parrinello, M., "Canonical sampling through velocity rescaling," *J Chem Phys* 126, no. 1 (2007), <https://doi.org/10.1063/1.2408420>.

[10]	Essmann, U., Perera, L., Berkowitz, M. L., et al., "A smooth particle mesh Ewald method," *The Journal of Chemical Physics* 103, no. 19 (1995), <https://doi.org/10.1063/1.470117>.

[11]	Humphrey, W., Dalke, A., Schulten, K., "VMD: Visual molecular dynamics," *Journal of Molecular Graphics* 14, no. 1 (1996), <https://doi.org/https://doi.org/10.1016/0263-7855(96)00018-5>.
